# Factors Affecting Public Adoption of COVID-19 Prevention and Treatment Information During an Infodemic: Cross-sectional Survey Study

**DOI:** 10.2196/23097

**Published:** 2021-03-11

**Authors:** Yangyang Han, Binshan Jiang, Rui Guo

**Affiliations:** 1 School of Public Health Capital Medical University Beijing China

**Keywords:** information adoption, infodemic, China, health information, infodemiology, COVID-19, public health

## Abstract

**Background:**

With the spread of COVID-19, an infodemic is also emerging. In public health emergencies, the use of information to enable disease prevention and treatment is incredibly important. Although both the information adoption model (IAM) and health belief model (HBM) have their own merits, they only focus on information or public influence factors, respectively, to explain the public’s intention to adopt online prevention and treatment information.

**Objective:**

The aim of this study was to fill this gap by using a combination of the IAM and the HBM as the framework for exploring the influencing factors and paths in public health events that affect the public’s adoption of online health information and health behaviors, focusing on both objective and subjective factors.

**Methods:**

We carried out an online survey to collect responses from participants in China (N=501). Structural equation modeling was used to evaluate items, and confirmatory factor analysis was used to calculate construct reliability and validity. The goodness of fit of the model and mediation effects were analyzed.

**Results:**

The overall fitness indices for the model developed in this study indicated an acceptable fit. Adoption intention was predicted by information characteristics (β=.266, *P*<.001) and perceived usefulness (β=.565, *P*<.001), which jointly explained nearly 67% of the adoption intention variance. Information characteristics (β=.244, *P*<.001), perceived drawbacks (β=–.097, *P*=.002), perceived benefits (β=.512, *P*<.001), and self-efficacy (β=.141, *P*<.001) jointly determined perceived usefulness and explained about 81% of the variance of perceived usefulness. However, social influence did not have a statistically significant impact on perceived usefulness, and self-efficacy did not significantly influence adoption intention directly.

**Conclusions:**

By integrating IAM and HBM, this study provided the insight and understanding that perceived usefulness and adoption intention of online health information could be influenced by information characteristics, people’s perceptions of information drawbacks and benefits, and self-efficacy. Moreover, people also exhibited proactive behavior rather than reactive behavior to adopt information. Thus, we should consider these factors when helping the *informed public* obtain useful information via two approaches: one is to improve the quality of government-based and other official information, and the other is to improve the public’s capacity to obtain information, in order to promote truth and fight rumors. This will, in turn, contribute to saving lives as the pandemic continues to unfold and run its course.

## Introduction

### Background

The global outbreak and rapid spread of the COVID-19 pandemic has led the world’s public health and safety systems to face great challenges. As of April 16, 2020, the COVID-19 pandemic has affected at least 211 countries, with 2,362,704 confirmed cases and more than 165,006 deaths globally, while China has confirmed 82,367 cases and the cumulative number of deaths was 3342 [1]. The disease rapidly spread in China because of the massive population migration (ie, Chunyun, also known as the Spring Festival travel rush, which refers to a human migration during this festival) and lack of prevention and control information during the early stage of the pandemic. Moreover, the World Health Organization (WHO) has not only signaled the health risks of COVID-19 but has also labeled the situation as an infodemic, due to the amount of information, both truth and rumors, circulating around this topic [2,3].

Although public health information dissemination represents an exciting combination of broadcasting, sharing, and retrieving relevant health information [4,5], the COVID-19 infodemic did not come as a surprise [6]. The massive growth of health information on the internet was seen to be a real problem [4]. Too much information makes it difficult to find trustworthy sources of information and may harm people’s health. Therefore, the quality of online health information is essential, especially when truth and rumors were still intertwined, which caused the infodemic. However, in the process of fighting COVID-19, the WHO and health authorities worldwide have been working closely with social media platforms, including Facebook, Google, Twitter, and YouTube, to provide evidence-based information to the general public in order to actively counter the rumors that have been circulating [7]. Prevention and treatment information about COVID-19 continues to spread and has an influence on populations.

At the same time, the lack of transparent, timely, and effective risk communication by health authorities regarding this emerging infectious disease in its early stage failed to bring about the appropriate level of public awareness and behavioral responses, such as avoidance of mass gatherings and personal protection in China, Europe, and the United States [8]. Online guidelines providing information for prevention and treatment are inconsistent in adapting to new knowledge, and changing or conflicting information can also confuse the public [9].

Since the beginning of the COVID-19 pandemic, information consumption has increased rapidly and significantly [10]. The new generation of health care consumers consists of an *informed* *public*, gleaning truth and rumors about health information with both positive and negative effects on themselves [11]. During periods of SARS spread, most people obtained SARS information from television [12]. By the time the Zika virus emerged in 2015-2016, Google Trends showed a significant increase in public searches on the internet related to the Zika virus [13], and the number of searches on video platforms, such as YouTube, had also increased rapidly [14]. Internet-based platforms that people utilized became diversified. During the COVID-19 pandemic, social distancing and stay-at-home restrictions caused the public to be fully exposed to social media; during this time, people actively searched or passively received a large amount of health-related information to prevent and treat diseases. In China, social media platforms, such as WeChat and Douyin (ie, TikTok), played an essential role in obtaining information after the virus began spreading [15]. However, studies have confirmed that over one-quarter of the most viewed YouTube videos about COVID-19 contained rumors, and these reached millions of viewers worldwide [16]. Therefore, determining whether people can use the prevention and treatment information they find on social media is critical in this pandemic.

Moreover, identifying health information from prevention and control measures is a major blind spot for the public. The public are partly responsible for selecting and filtering trustworthy health information [17]. Research shows that more than half of respondents trust almost all information online [18], and people are more likely to believe health rumors because of basic safety needs [19]. As the core part of health behavior theory, behavior intention is the subjective possibility of engaging in certain behavior. Some studies have confirmed that variables in the health belief model (HBM) can materially affect information adoption intention, and this process will affect subsequent health behaviors [20].

Therefore, if concern about identifying trustworthy information is reflected in the global population, we believe that the influencing factors in the subjective and objective aspects of engaging in that behavior may affect information adoption. Motivated by previous studies, this study was based on the information adoption model (IAM) and the HBM. The aim of this study was to explore the influencing factors and paths during public health events that affect the public’s intention to adopt online prevention and treatment information under the infodemic. We aim to provide a basis for decision making and policy suggestions in order to deal with online health information governance in the internet era. Moreover, this study adds to the sparse literature on information adoption.

### Research Model and Hypotheses

#### Information Adoption Model

Sussman and Siegal first proposed the IAM based on the technology acceptance model (TAM) and the theoretical perspective of the elaboration likelihood model (ELM), which regards how information influences people’s decision making as the process of information adoption [21]. From the TAM, a critical aspect of how individuals act on an advocated issue or behavior is the extent to which they believe the information contained within a message is useful [21,22]. From the ELM, this process depends on elaboration likelihood, and two likely antecedents of usefulness have been suggested from this stream of research as well as two key internal validity factors [21]. The ELM explains how individuals adopt information and then change their will and behavior. Moreover, recent literature has also demonstrated that this model can be applied in the context of online information acceptance, argument quality, and source credibility; these are taken as the direct objects of information adoption, and their influence has been repeatedly verified [23,24]. Simultaneously, individuals’ perceived information usefulness based on information quality and source characteristics plays a crucial intermediary role in information adoption. Health information as a type of information fits into the IAM’s influencing factors. The essence of information adoption is when individuals are persuaded by the received information and then accept the opinions or propositions expressed in the information.

#### Health Belief Model

The HBM has been widely used to explain preventive health behavior [25] and is one of the first and the best-known social cognition models. It focuses on the relationship of health behaviors, practices, and utilization of health services. From its initial design to predict behavioral response to the treatment received by chronic patients [26], it has been validated in different studies. Contemporary research studies have recently focused on the general health behavior of the population [27,28]. The core concept of HBM is people’s perception of disease threat and an assessment of their behavior. The assessment of behavior includes evaluating the effectiveness of behavior, the input and outcome of behavior change, and the obstacle to its implementation [28].

Furthermore, researchers added *cues to action*, meaning the stimuli or cues that catalyze action. Cues are divided into external cues, such as mass media, and internal cues, such as physical discomfort, that limit people’s beliefs about behavioral health consequences and behavioral effects. The HBM has been widely used in health behavior change [20], which provides scientific theoretical support for understanding the impact of health information propagation on the audience’s health behavior. It has become one of the most comprehensive models to understand health-related behaviors and why people undertake, or do not undertake, actions to prevent or control illnesses [29].

#### Integrated Model of IAM and HBM

Although IAM and HBM are commonly used models, the use of these models independently has not fully explained online health information adoption behavior. IAM focuses on the influence of information characteristics on information adoption without considering the individual’s subjective status quo. However, the information’s influence might change from person to person; the same content can evoke differing notions among receivers [30]. Also, HBM only considers the public’s cognitive information to predict general health behavior without influencing the process. More specifically, this study argues that an individual’s motivation to adopt health information will depend on the individual’s subjective and information-related objective factors. It is also supported by Erkan and Evans’ information acceptance model (IACM), in which a conceptual model was developed based on the integration of IAM and the theory of reasoned action; this model confirmed the influences of both information adoption and attitude toward information on consumers’ purchase intentions and the influence of information usefulness on information adoption [31]. In IACM, information quality, information credibility, and information needs were all found to affect information usefulness [31]. In addition, Ahadzadeh et al combined the TAM and HBM to study health-related internet use [32]. This study demonstrated that individuals who perceived their health to be at risk, or were motivated to use the internet when they believed that the internet was useful for providing health and health management information, would be expected to have a positive attitude toward internet use for health purposes [32].

**Figure 1:**
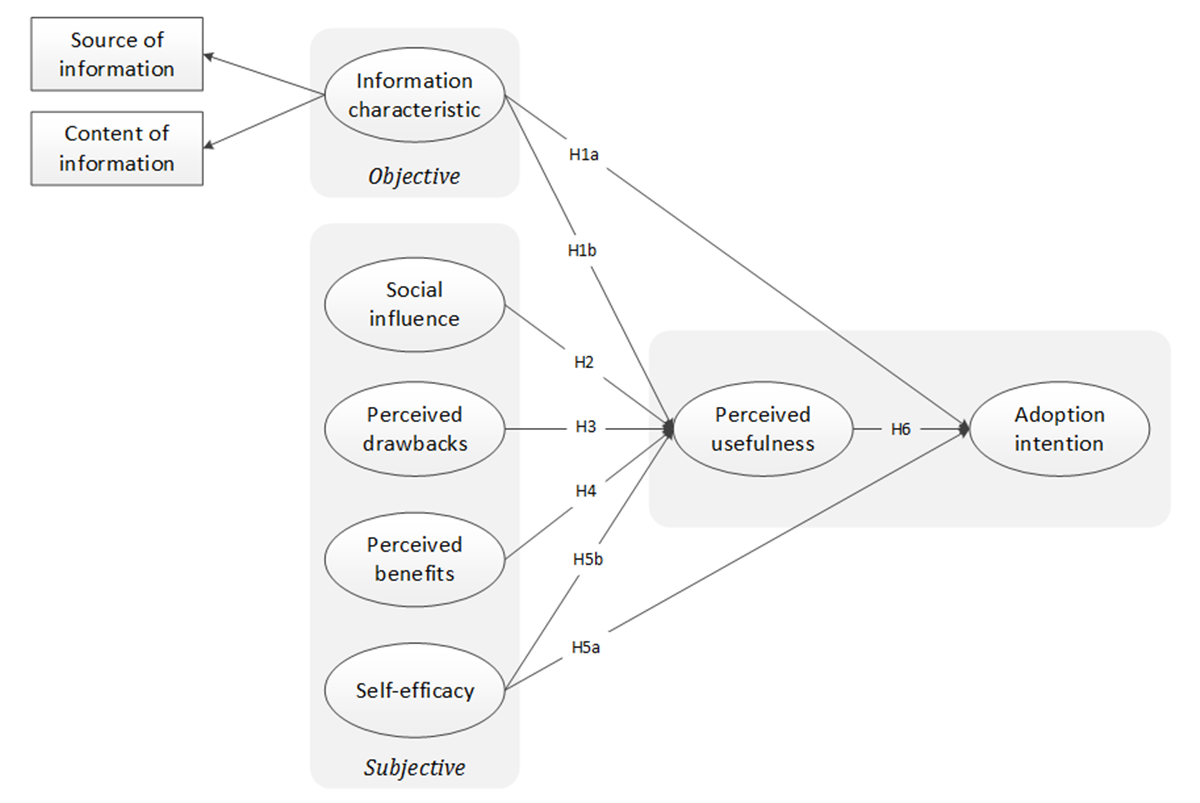
Integrated model based on the information adoption model and health belief model. H: hypothesis.

Our research mainly focused on information adoption behavior during the COVID-19 infodemic; therefore, we developed the IAM-HBM model by considering information characteristics (ie, objective factors) and the public’s health beliefs (ie, subjective factors) about information. Also, the HBM has been criticized for not considering environmental factors, such as social influence (ie, friends, family, and individuals’ internet providers), that can influence health-related behavior [33]. Therefore, we proposed the following path: social influence impacts perceived usefulness of information. Figure 1 shows the conceptual model proposed by this study, which incorporates information characteristics, social influence, perceived drawbacks, perceived benefits, self-efficacy, perceived usefulness, and adoption intention.

#### Hypotheses

*Information characteristics* refer to whether the prevention and treatment information about COVID-19 to which individuals are exposed from the internet is persuasive and to how individuals perceive the credibility of the information source, including the content and source attributes of the information. Sussman and Siegal believed that information characteristics, including quality and source, impacted people’s perceived information usefulness, which then affected information adoption; they also believed that information characteristics impacted individuals’ information adoption directly, which was measured by information adoption intention [21]. Thus, information characteristics are applied to our IAM-HBM model as verified factors in the IAM, impacting individuals’ perceived usefulness of COVID-19 prevention and treatment information. Based on this, we hypothesized the following:

1. Hypothesis 1a. Information characteristics are positively associated with the intention to adopt COVID-19 prevention and treatment information.
2. Hypothesis 1b. Information characteristics are positively associated with perceived usefulness of COVID-19 prevention and treatment information.

*Social influence* refers to how individuals perceive the influence of those around them when they adopt COVID-19 prevention and control information. Venkatesh et al defined social influence as the degree to which users were affected by people around them using new technologies and systems; this included integrated subjective norms, social factors, and images [34]. The construct of social influence was added to the extended HBM to enhance the prediction ability of the model. Perceived usefulness was mediated by external variables, including social influence [35,36].

*Perceived drawbacks* refer to the difficulties that individuals may encounter when adopting the prevention and treatment information of COVID-19 on the internet. Such difficulties become apparent by the predicted cost of adopting healthy behaviors, including tangible and intangible costs. Tangible cost refers to the cost of perceived usefulness of information, usually measured in monetary terms. Intangible cost refers to the effort required to confirm the usefulness of information, such as time and energy [37,38]. Based on HBM, perceived drawbacks were confirmed as the most powerful single predictor of intended expectations [39]. Yun built an integrated model and verified that perceived drawbacks affect people’s actions in seeking health information through perceived usefulness [40]. If social media users do not need to spend too much money or physical and mental energy, they can reduce the cost of using technology, and their total perceived usefulness will increase when searching for online health information [41].

*Perceived benefits* refer to the benefits that individuals may give themselves if they adopt the prevention and treatment information about COVID-19 on the internet. In the research on the HBM, Rosenstock proposed that individuals would weigh the effectiveness of behaviors through cost-benefit analysis when they adopt healthy behaviors. The perception of benefits provided a more preferential action path [41,42]. The positive experiences gained by individuals from acquiring and adopting health information behavior will promote the overall value perception of this behavior. This will further strengthen the intention of continuing to search for health information [41]. Building on findings by Rosenstock, we explored whether perceived benefits are associated with perceived usefulness. Thus, perceived benefits were applied to this IAM-HBM model as a factor that estimates individuals’ beliefs about the usefulness of COVID-19 prevention and treatment information.

*Self-efficacy* refers to the level of confidence in an individual’s ability to prevent and treat COVID-19. As an essential part of social cognitive theory, self-efficacy refers to people’s confidence in performing a specific behavior. The higher the expectations, the higher the tendency to make more considerable efforts. This concept has been widely used to understand health behavior change. Self-efficacy also had an impact on health information–seeking behavior [43]. Information on the internet regarding people’s health interventions significantly improved the specific behaviors of self-efficacy, including physical exercise and healthy eating. Physiological and social advantages caused people to have more positive behavior change expectations.

Yun’s integrated model demonstrated that internet self-efficacy affected users’ actions through perceived usefulness when seeking health information on the internet [40]. We gravitate toward the idea that self-efficacy is a determinant of adoption intention.

Based on the discussion above, we hypothesized the following:

1. Hypothesis 2. Social influence is positively associated with perceived usefulness of COVID-19 prevention and treatment information.
2. Hypothesis 3. Perceived drawbacks are negatively associated with perceived usefulness of COVID-19 prevention and treatment information.
3. Hypothesis 4. Perceived benefits are positively associated with perceived usefulness of COVID-19 prevention and treatment information.
4. Hypothesis 5a. Self-efficacy is positively associated with COVID-19 prevention and treatment information adoption.
5. Hypothesis 5b. Self-efficacy is positively associated with perceived usefulness of COVID-19 prevention and treatment information.

*Perceived usefulness* refers to an individual’s blanket perception of COVID-19 prevention and treatment information on the internet. It is plausible that adopting such healthy behaviors can meet individuals’ health needs and help them achieve healthy outcomes. Usefulness, utility, and perceived usefulness, which were first applied to TAM by Davis et al, are used to evaluate the utility of information-seeking behavior [22]. As the crucial variable of IAM, perceived usefulness has a significant influence on adoption intention. Thus, perceived usefulness is applied to this IAM-HBM model as a factor that could influence individuals’ COVID-19 prevention and treatment information adoption. In our paper, Hypothesis 6 states that perceived usefulness is positively associated with the intention to adopt COVID-19 prevention and treatment information.

## Methods

### Data Collection and Participants

Online questionnaires were powered by the survey platform WJX (Changsha Ranxing Information Technology Co), whose web application was embedded into social media platforms from March 24 to April 5, 2020. The electronic version of the questionnaire was uploaded to the WJX web application; respondents (ie, Chinese people in China) could fill in, submit, and share the questionnaire using a Quick Response (QR) code or using a forwarding link issued by the WJX web application. The data were collected using snowball sampling through repetitive one-to-many sharing on social media, a nonprobability sampling method; there were 528 respondents from 30 provinces in China. We gathered data using an online survey because of public space restrictions and because netizens were potentially exposed to the infodemic. After eliminating invalid responses through data filtering, 501 valid questionnaires out of 528 remained (94.9% validity rate). Table 1 shows the demographic characteristics of the sample population.

**Table 1:** Demographic characteristics of participants.

Characteristic		Value (N=501), n (%)
**Gender**		
	Male	216 (43.1)
	Female	285 (56.9)
**Age (years)**		
	18-19	6 (1.2)
	20-29	154 (30.7)
	30-39	122 (24.4)
	40-49	81 (16.2)
	50-59	77 (15.4)
	60-69	51 (10.2)
	70-79	8 (1.5)
	≥80	2 (0.4)
**Education**		
	Junior high school diploma or below	21 (4.2)
	Senior high school diploma	66 (13.2)
	College graduate	84 (16.8)
	Bachelor’s degree	256 (51.1)
	Master’s degree or above	74 (14.8)
**Occupation**		
	Student	104 (20.8)
	Officer^a^	50 (10.0)
	Enterprise manager	67 (13.4)
	Office staff or clerk	62 (12.4)
	Professional^b^	75 (15.0)
	Worker or laborer	24 (4.8)
	Business service	14 (2.8)
	Self-employed	12 (2.4)
	Freelancer	22 (4.4)
	Farmer	2 (0.4)
	Retired	63 (12.6)
	No profession^c^	6 (1.2)

^a^Officer occupations include government officials, cadres, and civil servants.

^b^Professional occupations include doctors, lawyers, journalists, teachers, etc.

^c^No profession includes temporary occupation or unemployed.

Out of the 501 valid responses, 216 (43.1%) were from male participants and the other 285 (56.9%) were from female participants. Further, the majority of respondents were between the ages of 20 and 39 years (276/501, 55.1%). Most of the respondents had earned a bachelor’s degree (256/501, 51.1%), which indicated a high level of education among respondents. In terms of occupation, the largest group was students (104/501, 20.8%), followed by professionals (75/501, 15.0%) and enterprise managers (67/501, 13.4%).

### Quality Control

We conducted quality control through the survey platform and via the investigators. Once on the platform, respondents were invited to fill in the questionnaire voluntarily. Each Internet Protocol (IP) address, computer, or username could only be used once. Also, there were various filtering rules for invalid answers, such as spending too little time on a questionnaire and trap rules to filter out random answers.

After the surveys were submitted, the respondents were screened by the investigators to retain the valid questionnaires. We excluded the following respondents: those who failed the attention check, where the answers to all the questions were the same or cyclical; those with response times of less than 120 seconds; those under 18 years old; and non-Chinese residents. Also, we checked the consistency between the IP address and the selected region, and questionnaires were eliminated if the IP addresses were not consistent.

### Measures

The study instrument was modified from those in the relevant existing literature. Measurements and scales were translated into the appropriate Chinese versions to ensure the completeness and accuracy of instruments. After the repeated pretest, the final questionnaire was translated back into English, and the main semantics were not changed, indicating a strong correlation with the original English questionnaire (see Table 2 [21,29,34,44-52]). The instruments were measured using a 5-point Likert scale, ranging from 1 (highly disagree) to 5 (highly agree). All dimensions included three items, except the information characteristics construct, which included five items.

**Table 2:** Measurement items of the constructs.

Construct and variables		Measurement item
**Information characteristics (IC) [21,45,46]**		
	IC1	COVID-19 prevention and treatment information on the internet is appropriate for my health demands.
	IC2	COVID-19 prevention and treatment information on the internet is understandable.
	IC3	COVID-19 prevention and treatment information on the internet is shared by most people (eg, by thumb-up or retweet).
	IC4	The argument for COVID-19 prevention and treatment information on the internet is compelling.
	IC5	The publisher of COVID-19 prevention and treatment information on the internet is experienced in the health field.
**Social influence (SI) [44,47]**		
	SI1	People who are important to me think I should get COVID-19 prevention and treatment information from the internet.
	SI2	My family and friends have obtained COVID-19 prevention and treatment information from the internet.
	SI3	It is prevalent to get COVID-19 prevention and treatment information from the internet.
**Perceived drawbacks (PD) [48,49]**		
	PD1	It may take me too much time or expense to adopt COVID-19 prevention and treatment information from the internet.
	PD2	Adopting COVID-19 prevention and treatment information from the internet may cause psychological stress.
	PD3	The health risks associated with the adoption of COVID-19 prevention and treatment information from the internet may outweigh the positive health outcomes.
**Perceived benefits (PB) [29]**		
	PB1	It is important for me to adopt COVID-19 prevention and treatment information to reduce my risk of COVID-19 infection.
	PB2	Adopting COVID-19 prevention and treatment information can help me stay healthy, which is very important to me.
	PB3	The adoption of COVID-19 prevention and treatment information is valuable for me in order to adopt COVID-19 prevention behaviors.
**Self-efficacy (SE) [50]**		
	SE1	I am confident that I can avoid COVID-19.
	SE2	I can figure out how to avoid COVID-19 infection.
	SE3	Even if I contract COVID-19, I can recover soon.
**Perceived usefulness (PU) [46,51]**		
	PU1	The COVID-19 prevention and treatment information on the internet is valuable to me in preventing COVID-19.
	PU2	I can make good use of COVID-19 prevention and treatment information on the internet in my life.
	PU3	COVID-19 prevention and treatment information on the internet can improve the health of my family, friends, and myself.
**Adoption intention (AI) [34,52]**		
	AI1	I will recommend this COVID-19 prevention and treatment information to my family and friends.
	AI2	I will use this COVID-19 prevention and treatment information obtained from the internet in my daily life.
	AI3	I would like to adopt COVID-19 prevention and treatment information, even if it takes my time or money (ie, to buy drugs, protective equipment, etc) to do so.

### Ethics Approval and Consent to Participate

This study was approved in writing by the Medical Ethics Committee of Capital Medical University (No. Z2019SY014) and all participants gave informed consent.

## Results

### Overview

After data collection, the two-stage procedure of structural equation modeling (SEM) was applied to conduct data analysis [41]. The first procedure examined scale validity from the measurement model by confirmatory factor analysis (CFA), while the second procedure interpreted hypotheses testing by the structural model. Both SPSS Statistics for Windows, version 19.0 (IBM Corp), and SPSS Amos, version 24.0 (IBM Corp), were adopted as the tools for analyzing the data.

### Measurement Model

#### Reliability

In this study, questionnaire items had a factor loading of 0.592 and above (see Table 3), which met the evaluation standard that the factor loading for construct measures must exceed 0.5 to be retained [53]. Cronbach α should be at least .70, and high reliability is assumed if it is greater than .80 [54]. The composite reliability (CR) value of greater than 0.70 represented high reliability [53]. All the constructs had both high Cronbach α and CR values, indicating high reliability (see Table 3).

#### Convergent Validity

Convergent validity measures the correlation of a dimension’s multiple indicators. This study used the average variance extracted (AVE) to estimate the convergent validity [53]. A dimension with an AVE value over 0.50 would be considered as having high convergent validity [55]. As shown in Table 3, all dimensions had AVE values that were higher than the aforementioned cutoff values, which suggest good convergent validity.

In addition, all factor loadings for indicators measuring the same construct were statistically significant (see Table 3), suggesting that all indicators effectively measured their corresponding construct [56] and supported convergent validity.

**Table 3:** Reliability and convergent validity.

Construct and scale items		Factor loading^a^	Cronbach α^b^	Composite reliability coefficient^b^	Average variance extracted^b^
**Information characteristics (IC)**			.903	0.904	0.654
	IC1	0.822	—	—	—
	IC2	0.765	—	—	—
	IC3	0.858	—	—	—
	IC4	0.840	—	—	—
	IC5	0.754	—	—	—
**Social influence (SI)**			.834	0.841	0.642
	SI1	0.702	—	—	—
	SI2	0.926	—	—	—
	SI3	0.758	—	—	—
**Perceived drawbacks (PD)**			.753	0.756	0.510
	PD1	0.633	—	—	—
	PD2	0.789	—	—	—
	PD3	0.712	—	—	—
**Perceived benefits (PB)**			.894	0.897	0.745
	PB1	0.835	—	—	—
	PB2	0.930	—	—	—
	PB3	0.820	—	—	—
**Self-efficacy (SE)**			.773	0.788	0.558
	SE1	0.837	—	—	—
	SE2	0.790	—	—	—
	SE3	0.592	—	—	—
**Perceived usefulness (PU)**			.884	0.885	0.719
	PU1	0.826	—	—	—
	PU2	0.869	—	—	—
	PU3	0.849	—	—	—
**Adoption intention (AI)**			.868	0.875	0.702
	AI1	0.831	—	—	—
	AI2	0.944	—	—	—
	AI3	0.723	—	—	—

^a^All factor loadings were significant at the *P*<.001 level.

^b^This value was calculated for each construct and not for individual items.

#### Discriminant Validity

Discriminant validity is achieved if the correlations between different constructs are relatively significant. The chi-square difference test can assess the discriminant validity of every two constructs by calculating the difference of the chi-square statistics for the constrained and unconstrained measurement models [57]. In this study, except for perceived drawbacks, the other six dimensions’ chi-square difference tests were significant at the *P*=.05 level (see Table 4). Accordingly, the results demonstrated that discriminant validity was successfully achieved for the measurement model.

**Table 4:** Correlation analysis among constructs to determine discriminant validity.^a^

Construct	IC^b^	SI^c^	PD^d^	PB^e^	SE^f^	PU^g^	AI^h^
IC	0.809	—^i^	—	—	—	—	—
SI	0.729	0.801	—	—	—	—	—
PD	–0.044	–0.040	0.714	—	—	—	—
PB	0.741	0.799	–0.068	0.863	—	—	—
SE	0.437	0.421	0.023	0.512	0.747	—	—
PU	0.773	0.761	–0.152	0.857	0.555	0.848	—
AI	0.723	0.664	–0.038	0.741	0.479	0.792	0.838

^a^Diagonal elements are the square root of average variance extracted of the reflective scales. Off-diagonal elements are correlations between constructs.

^b^IC: information characteristics.

^c^SI: social influence.

^d^PD: perceived drawbacks.

^e^PB: perceived benefits.

^f^SE: self-efficacy.

^g^PU: perceived usefulness.

^h^AI: adoption intention.

^i^Repeated values were not included for easier comparison of table values.

Suppose the absolute value of the correlation coefficient is less than the square root of the AVE value. That would indicate that each construct has a certain correlation and a certain degree of differentiation between constructs, indicating that the scale data have an ideal discriminant validity. The value of the AVE square root of each construct was greater than the square of its correlation coefficient with the dimensions of all dimensions.

### Structural Model Analysis

Based on the results of the CFA and modification index of indicator variables, six standard model fit criteria were used to assess the model’s overall goodness of fit: ratio of the chi-square value to the degrees of freedom (χ^2^/df), goodness-of-fit index (GFI), comparative fit index (CFI), Tucker-Lewis index (TLI), root mean square residual, and root mean square error of approximation (RMSEA).

As shown in Table 5, comparison of all fit indices with their corresponding recommended values provided evidence of a good model fit: χ^2^/df values were between 1.0 and 3.0; GFI, CFI, and TLI were all greater than 0.9; and RMSEA was smaller than 0.08. This demonstrated that the measurement model exhibited a tolerably good fit with the data collected [56].

**Table 5:** Goodness of fit of the measurement and structural models.

Statistical check	Goodness-of-fit criteria	Measurement model	Structural model	Result
χ^2^/df	1.0-3.0	2.685	2.677	Good
Goodness-of-fit index	>0.9	0.908	0.908	Good
Comparative fit index	>0.9	0.953	0.953	Good
Tucker-Lewis index	>0.9	0.944	0.944	Good
Root mean square error of approximation	<0.08	0.058	0.058	Pass

### Structure Model

Based on the results of SEM, the fit indices of the structural model are shown in Table 5. Under the same criteria, the structure model fits the observed data as well. Meanwhile, the estimated results of the structural model provided the path coefficients shown in Table 6. Among the eight hypotheses, six paths were supported based on the valid data, significant at the *P*=.01 level, while the remaining two paths were rejected according to SEM (ie, Hypothesis 2 and Hypothesis 5a).

**Figure 2:**
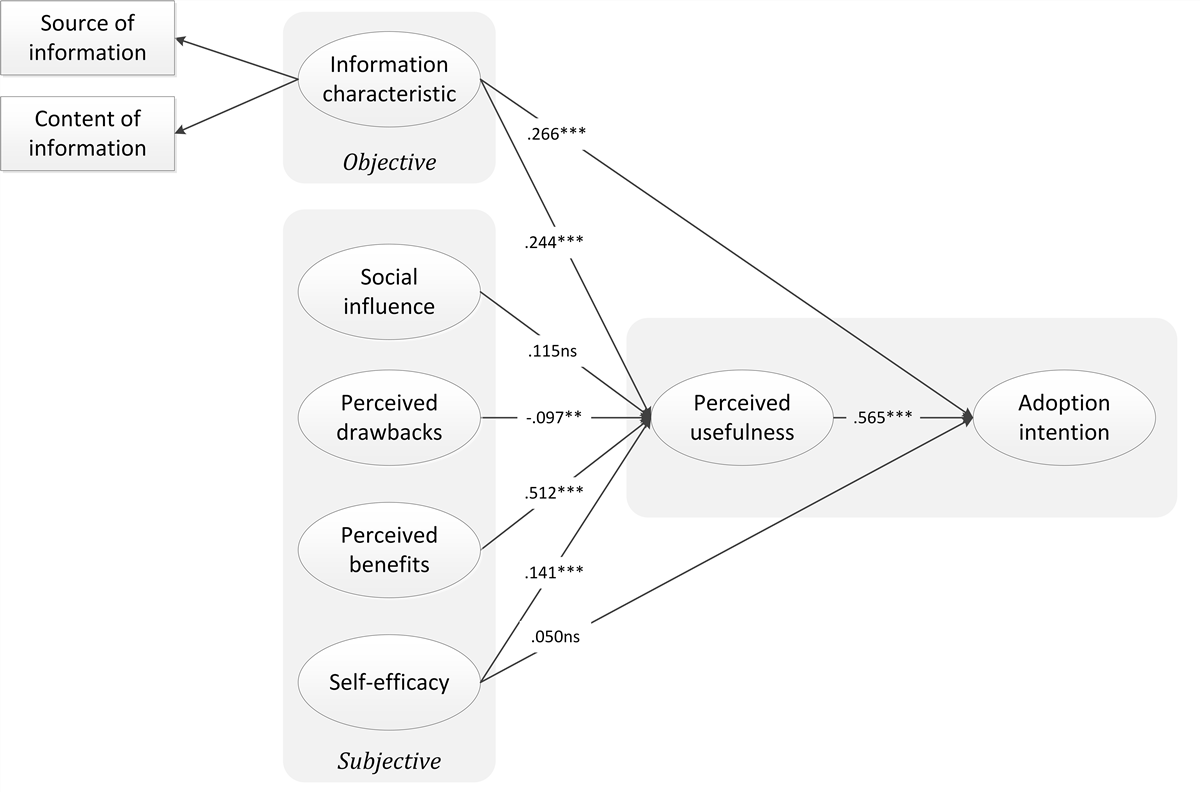
Structural model results; standardized path coefficients (β) are shown. ***P<.001; **P<.01; ns: not significant (*P*>.05).

Adoption intention was predicted by information characteristics (β=.266, *P*<.001) and perceived usefulness (β=.565, *P*<.001), which jointly explained 66.8% of the adoption intention variance. Information characteristics (β=.244, *P<.*001), perceived drawbacks (β=–.097, *P*=.002), perceived benefits (β=.512, *P*<.001), and self-efficacy (β=.141, *P*<.001) jointly determined perceived usefulness and explained 81.1% variance of perceived usefulness. In addition, perceived usefulness had the most significant influence on adoption intention. It revealed that perceived usefulness was an important indicator of adoption intention. Perceived benefits had the most significant direct influence on perceived usefulness, followed by information characteristics, while perceived drawbacks had a relatively low path coefficient, which indicated a negative effect at the same time. Since the rejection of Hypothesis 5a, it means that perceived usefulness had a complete mediating effect on self-efficacy to adoption intention. This result confirmed perceived usefulness as an intermediary variable. Surprisingly, Hypothesis 2 was not supported in this study. The path coefficients supported six of all hypothesized relationships (see Table 6, Figure 2, and Multimedia Appendix 1).

**Table 6:** Testing results of the hypotheses.

Hypothesis	Path	Standardized path coefficient (β)	*P* value	Result
Hypothesis 1a	IC^a^→PU^b^	0.244	<.001	Supported
Hypothesis 1b	IC→AI^c^	0.266	<.001	Supported
Hypothesis 2	SI^d^→PU	0.115	.07	Rejected
Hypothesis 3	PD^e^→PU	–0.097	.002	Supported
Hypothesis 4	PB^f^→PU	0.512	<.001	Supported
Hypothesis 5a	SE^g^→PU	0.141	<.001	Supported
Hypothesis 5b	SE→AI	0.050	.25	Rejected
Hypothesis 6	PU→AI	0.565	<.001	Supported

^a^IC: information characteristics.

^b^PU: perceived usefulness.

^c^AI: adoption intention.

^d^SI: social influence.

^e^PD: perceived drawbacks.

^f^PB: perceived benefits.

^g^SE: self-efficacy.

### Analysis of Mediating Effects

To test the indirect effects, bias-corrected bootstrapping with 5000 iterations was implemented to obtain the structural model path significance levels for indirect effects [58]. Bootstrapping, a nonparametric approach, was superior to other approaches in testing mediation models because it does not assume multivariate normality [59,60]. Table 7 shows that perceived usefulness fully mediated the effect of perceived drawbacks, perceived benefits, and self-efficacy on adoption intention, whereas perceived usefulness partially mediated the effect of information characteristics on adoption intention.

**Table 7:** Mediation effect analysis.

Constructs of measurement	Standardized indirect effect	Bias-corrected values		Percentile		Mediating effect	
		95% CI	*P* value	95% CI	*P* value		
IC^a^→PU^b^→AI^c^	0.138	0.069 to 0.218	<.001	0.071 to 0.220	<.001	Partial	
SI^d^→PU→AI	0.065	–0.014 to 0.150	.10	–0.020 to 0.145	.13	No	
PD^e^→PU→AI	–0.055	–0.094 to –0.023	.002	–0.091 to –0.020	.004	Fully	
PB^f^→PU→AI	0.289	0.183 to 0.426	<.001	0.178 to 0.419	<.001	Fully	
SE^g^→PU→AI	0.079	0.026 to 0.152	.002	0.026 to 0.152	.002	Fully	

^a^IC: information characteristics.

^b^PU: perceived usefulness.

^c^AI: adoption intention.

^d^SI: social influence.

^e^PD: perceived drawbacks.

^f^PB: perceived benefits.

^g^SE: self-efficacy.

## Discussion

### Principal Findings

This study set out to determine the factors affecting people’s intention of adopting health information on the internet, and the IAM-HBM model was applied. In general, this combined model provided an excellent fit to the data. Our research supports perceived drawbacks, perceived benefits, self-efficacy, perceived usefulness, and information characteristics as factors associated with the intention to adopt online prevention and treatment information to prevent an epidemic in the context of COVID-19.

### Evidence-Based Information Plays an Important Role

In the study, information characteristics can strengthen perceived usefulness to adopt health information. In addition, the quality and the source of information influence the perceived usefulness and indirectly impact adoption intention. People are inclined to take in evidence-based information rather than misinformation on the internet [61,62]. Therefore, health communication should include more evidence-based information and should meet the public’s health demand [7]. Swamped by information on the internet, if the information is expressed more reliably or if its publisher is more professional and authoritative, the public will have a more robust perception of the usefulness of the information, thus increasing individuals’ willingness to adopt the information. At the same time, in the face of information overload, especially where some information constituted rumors, the public, who lack professional knowledge, will balance the outcomes of adoption information behavior. Many online repositories full of valuable content are underutilized, becoming “information junkyards” [63], and during the COVID-19 pandemic, an infodemic could be triggered by rumors. As information flow has improved, infodemic prevention and management using facts and evidence can mitigate the next infodemic [64,65].

### Improving the Capacity to Obtain Public Information to Fight Mixed Messages

The public’s perceived drawbacks, perceived benefits, and self-efficacy had significant influences on perceived usefulness. Members of the public conduct a cost-benefit analysis before adopting healthy behaviors, in which they weigh the effectiveness of the adoption against the possible cost and risk of time-consuming impediments. As a result, the more benefits and fewer drawbacks one perceives, the more that the health benefits of the adoption behavior outweigh the health risks, resulting in higher perceived usefulness by individuals in considering online health information to prevent COVID-19.

However, only by improving the public’s media literacy and their ability to perceive information can they correctly recognize the obstacles and benefits. At the same time, greater health literacy can improve public health self-efficacy, resulting in increased confidence in adopting healthy behaviors or changing bad behaviors. Many people have limited health literacy [66]; health communication and education are the most cost-effective means to improve health literacy [67]. Therefore, in the release of COVID-19-related health information, attention should be paid to improving public information capacity. For example, officials could actively hold health lectures and disclose health information, and relevant experts could improve the public’s capacity regarding obtaining information.

Simultaneously, with the continuous enrichment of social media, it is difficult for social communication based on social media to achieve full, comprehensive, and balanced transmission of information. We need to avoid falling into information cocoons and confirmation bias [68], and we need to measure the quality of information from an overall perspective.

### Government Has Greater Influence Than Family and Friends

Surprisingly, our analysis did not support the hypothesis that social influence is positively associated with perceived usefulness of COVID-19 prevention and treatment information. This finding was counterintuitive, and previous research showed that social networks positively affect people by encouraging them to adopt different health behavior intentions [69].

We think that government press conferences and news-based public opinion during the COVID-19 pandemic in China have weakened social influence on people’s perception and acceptance of health information for the following reasons. First, since the SARS epidemic in 2003, the Chinese government has reformed the news release concept and system. In response to the emergencies, the Chinese government issued a series of policy documents and established the State Council Information Office’s three-level news spokesman system for all central ministries and provincial-level people’s governments [70]. Second, governments at all levels use various channels to publicize, in a timely manner, the prevention and treatment information regarding COVID-19, the latest situation regarding the pandemic, and other public concerns, providing the public with a low threshold and low-cost direct information feedback channel [71]. The government also invited medical experts, such as Dr Zhong Nanshan, Head of the National Team for Control of Novel Coronavirus, to communicate with the public, and this strategy gained public trust [72]. Finally, China’s political system practices high-quality and high-efficiency unified decision making, and they have strict controls over content such as social media [73]. For example, Facebook, Twitter, and YouTube are not allowed in mainland China [74], and information monitoring and timely rumor controls are also available within popular social media platforms, such as WeChat and Sina Weibo [75]. Therefore, the government exerted its full influence during the pandemic to position itself as the primary influence [76].

However, social media is both a source of the infodemic and a public health tool [77]. Therefore, it is necessary to include social media platforms in public information dissemination; rethinking the role of public communication will also be necessary to assume corresponding responsibility during the pandemic. The responsibility is not only to *delete* information but, as much as possible, to ensure the diversity of the information environment to a sufficient degree; this will enable high-quality public content and thereby increase public participation [64].

### Conclusions

In a public health emergency, the online infodemic forces the public to negotiate with prevention and treatment information. By integrating IAM and HBM, this study provided the insight and understanding that perceived usefulness and adoption intention of online health information could be influenced by information characteristics, people’s perceptions of drawbacks and benefits, and self-efficacy. Moreover, people also exhibit proactive behavior rather than reactive behavior. Thus, we should consider these factors to help the *informed public* obtain useful information via two approaches: one is to control the quality of information and the other is to improve the public’s capacity to obtain information, in order to promote trusted information and to fight misinformation. This will, in turn, contribute to saving lives as the pandemic continues to unfold and run its course.

### Limitations

We administered the questionnaire survey during the stage of the pandemic in mainland China when it was under control, which was when the outbreak in China had passed the initial panic stage. People at different stages of the pandemic may have been influenced differently by the influencing factors. Therefore, although we found that social influence had no significant effect on perceived usefulness of information, a more comprehensive future study is suggested to explore whether this is due to social context, stage of the pandemic, or other factors. Moreover, this cross-sectional study was conducted using the WJX web application, and sample populations had a certain amount of experience in filling out online questionnaires and internet use. Therefore, a more comprehensive future study is suggested to include offline and online participants to expand the framework’s application scope.

## Appendix

Multimedia Appendix 1 Path diagram of the information adoption model (IAM)–health belief model (HBM).

## Abbreviations

AVE: average variance extracted
CFA: confirmatory factor analysis
CFI: comparative fit index
CR: composite reliability
ELM: elaboration likelihood model
GFI: goodness-of-fit index
HBM: health belief model
IACM: information acceptance model
IAM: information adoption model
IP: Internet Protocol
QR: Quick Response
RMSEA: root mean square error of approximation
SEM: structural equation modeling
TAM: technology acceptance model
TLI: Tucker-Lewis index
WHO: World Health Organization
