# Supplementary figures and images for: Factors Affecting Public Adoption of COVID-19 Prevention and Treatment Information During an Infodemic: Cross-sectional Survey Study
Source: J Med Internet Res. 2021 Mar 11;23(3):e23097. doi: 10.2196/23097 (PMC7954112; doi:10.2196/23097)

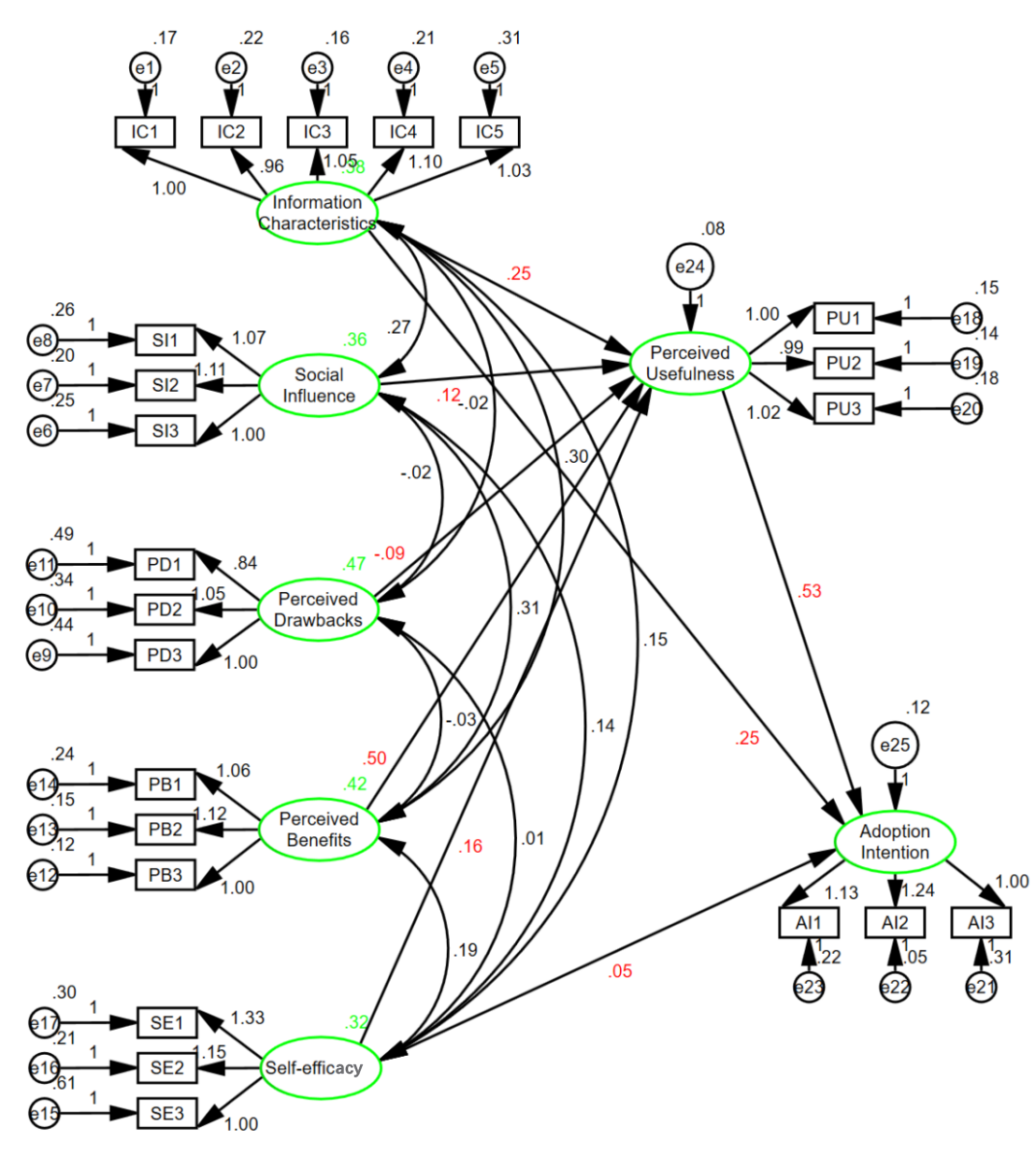

Supplement: Multimedia Appendix 1 [file jmir_v23i3e23097_app1.png]
